# Metabolic profile and skeletal muscle as predictors of survival in testicular germ cell tumors

**DOI:** 10.1093/oncolo/oyag072

**Published:** 2026-04-16

**Authors:** Alejandra Barrón-Hernández, Juan Alberto Ríos-Rodríguez, José Antonio García-Pacheco, Berenice Cuevas-Estrada, Sebastián De-la-Rosa, Clementina Castro-Hernández, Miguel A Jiménez-Ríos, Nora Sobrevilla-Moreno, Miguel Santibáñez-Andrade, Rodrigo González-Barrios

**Affiliations:** Laboratorio de Regulación de la Cromatina y Genómica, Instituto Nacional de Cancerología, Mexico City, 14080, Mexico; Laboratorio de Regulación de la Cromatina y Genómica, Instituto Nacional de Cancerología, Mexico City, 14080, Mexico; Laboratorio de Regulación de la Cromatina y Genómica, Instituto Nacional de Cancerología, Mexico City, 14080, Mexico; Laboratorio de Regulación de la Cromatina y Genómica, Instituto Nacional de Cancerología, Mexico City, 14080, Mexico; Laboratorio de Regulación de la Cromatina y Genómica, Instituto Nacional de Cancerología, Mexico City, 14080, Mexico; Laboratorio de Regulación de la Cromatina y Genómica, Instituto Nacional de Cancerología, Mexico City, 14080, Mexico; Departamento de Urología, Instituto Nacional de Cancerología, Mexico City, 14080, Mexico; Departamento de Oncología Médica, Instituto Nacional de Cancerología, Mexico City, 14080, Mexico; Laboratorio de Regulación de la Cromatina y Genómica, Instituto Nacional de Cancerología, Mexico City, 14080, Mexico; Departamento de Biología Celular, Facultad de Ciencias, UNAM, Mexico City, 04510, Mexico; Laboratorio de Regulación de la Cromatina y Genómica, Instituto Nacional de Cancerología, Mexico City, 14080, Mexico; Departamento de Biología Celular, Facultad de Ciencias, UNAM, Mexico City, 04510, Mexico

**Keywords:** testicular cancer, TGCT, body mass index, lean mass index, albumin, cancer survival

## Abstract

**Background:**

Testicular germ cell tumors (TGCT) are highly curable malignancies, yet the prognostic impact of host metabolic status remains underexplored, especially in non-Caucasian populations.

**Methods:**

We analyzed 2755 Mexican patients with TGCT treated at the Instituto Nacional de Cancerología (2007-2021). A Metabolic sub-cohort (*n* = 586) and an Imaging sub-cohort (*n* = 231) with baseline CT-derived lean mass index (LMI) were evaluated. Statistical analyses included multivariate Cox regression adjusted for IGCCCG risk, principal component analysis (PCA)-based clustering, and internal validation using bootstrapping.

**Results:**

Multivariate Cox regression adjusted for IGCCCG risk identified LMI (HR 0.95, 95% CI 0.90-0.99, *P* = .047), serum albumin (HR 0.27, 95% CI 0.18-0.42, *P* < .001), and HDL cholesterol (HR 0.94, 95% CI 0.91-0.96, *P* < .001) as independently associated factors. PCA-based metabolic-nutritional risk profiles effectively stratified 5-year survival in patients with non-seminoma (from 16.1% in depleted profiles to 97.4% in preserved profiles) and seminoma (64.0% vs 100%). A reduced prognostic model (BMI, LMI, albumin) achieved a robust optimism-corrected AUC of 0.866.

**Conclusions:**

Metabolic and body composition profiling provides prognostic discrimination complementary to the IGCCCG classification, suggesting the potential utility of incorporating objective nutritional assessments. Early identification of patients with metabolic vulnerability—specifically hypoalbuminemia, skeletal muscle depletion, and altered lipid profiles—could help guide personalized supportive strategies prioritizing nutritional and medical optimization to improve outcomes in TGCT.

## Graphical abstract

**Graphical Abstract:**
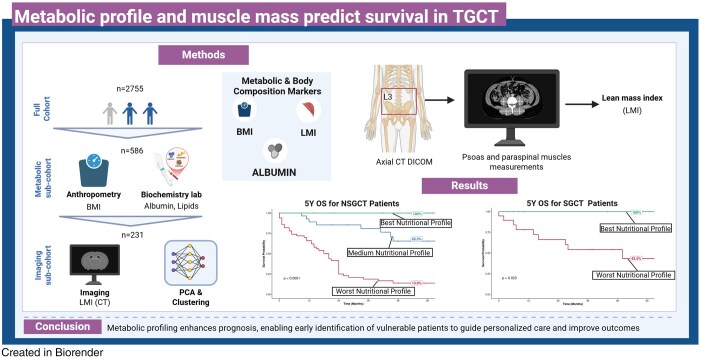

Implications for Practice This study proposes an integrated risk approach for TGCT by combining metabolic profiling and body composition assessment Our findings suggest that a high-risk metabolic profile—characterized by low BMI, skeletal muscle depletion, hypoalbuminemia, and an altered lipid profile—may provide prognostic information complementary to the standard IGCCCG risk group. Incorporating these accessible markers into routine clinical practice could enhance risk stratification by identifying vulnerable patients who may benefit from closer monitoring. This approach supports a shift toward prioritizing early supportive interventions and nutritional optimization, aimed at improving patient resilience and care throughout the oncologic treatment trajectory.

## Introduction

Testicular cancer is the most common solid tumor in young men and is generally considered a highly curable malignancy. However, in Mexico, mortality rates are nearly 5 times higher than the global average, a disparity that persists despite protocol-based therapy.^1–3^ Prognosis in testicular germ cell tumors (TGCT) is linked to histology, with seminomas (SGCT) having a more favorable outcome than the more aggressive non-seminomatous (NSGCT) subtypes.^1^^,^^4^

Growing evidence suggests that traditional factors like clinical stage and histology do not fully account for outcome disparities, particularly in Mexican cohorts.^5–8^ This highlights the need to incorporate host-related factors into prognostic models. Metabolic and nutritional status, reflected by body mass index (BMI), serum biochemical markers, and imaging-based body composition, have emerged as key determinants of prognosis in oncology but remain underexplored in TGCT.^9–12^

Previous studies have typically assessed these markers in isolation, such as BMI or albumin alone, without creating an integrated prognostic model that integrates body composition, lipids, and nutritional status.^10^^,^^11^^,^^13^^,^^14^ This gap is especially critical in Latin American patients, where metabolic profiles may differ and markers like albumin, lipids, and muscle mass—all linked to inflammation and treatment tolerance—are yet to be fully investigated.^6^^,^^8^^,^^15^^,^^16^

This study aims to explore the prognostic value of baseline BMI, lean mass index (LMI), albumin, and lipids on 5-year overall survival (OS) in Mexican patients with TGCT. By integrating clinical, biochemical, and imaging data, using principal component analysis (PCA) and clustering we seek to delineate exploratory metabolic-nutritional risk profiles complementary to conventional classifications.^17^^,^^18^

## Methods

### Study design and population

We conducted a single-center, retrospective cohort study at the National Cancer Institute (INCan) of Mexico City (2007-2021) using a nested design with 3 tiers. The full cohort included patients with histopathologically confirmed TGCT. The metabolic sub-cohort was formed by patients with pre-chemotherapy fasting biochemical profiles, from which an imaging sub-cohort with suitable baseline CT scans was derived.

### Eligibility criteria

Inclusion in the full cohort required complete baseline records and follow-up. The metabolic sub-cohort required biomarkers collected ≤14 days before chemotherapy. The imaging sub-cohort required CT scans performed ≤30 days pre-chemotherapy with adequate L3 visualization; scans with significant artifacts were excluded (Figure S1, see online supplementary material for a color version of this figure).

### Clinical management and treatment

Treatment at our institution followed a uniform standard of care based on NCCN guidelines. Patients in the imaging sub-cohort received standard cisplatin-based regimens (eg, BEP x3/x4 or EP x4) dictated directly by their prognostic group.

### Variable and outcomes

Baseline demographic, anthropometric (weight, height, BMI), and metabolic variables (fasting albumin, glucose, lipids) were collected at diagnosis (Table 1).

**Table 1.** Baseline demographic and clinical characteristics of patients with TGCT stratified by vital status.

Variable	Total	Alive	Deceased	*P*-value
	(*n* = 2755)	(*n* = 2343)	(*n* = 412)	
**Age, years (median) (Q1-Q3)**	27 (22-32)	27 (22-32)	26 (21-31)	.0307
**Body weight, kg (median) (Q1-Q3)**	72 (64-80.5)	72 (64-81.5)	70 (61-78)	<.001
**Height, cm (median) (Q1-Q3)**	1.7 (1.65-1.74)	1.7 (1.65-1.74)	1.7 (1.64-1.73)	<.001
**Body mass index (BMI), kg/m^2^ (median) (Q1-Q3)**	24.9 (22.6-27.7)	25.1 (22.7-28)	24.2 (22.075-26.8)	<.001
**Clinical stage of disease^a^**				
**Stage I (localized) (%)**	658	645 (27.5%)	13 (3.2%)	
**Stage II (regional lymph node involvement) (%)**	351	320 (13.6%)	31 (7.5%)	
**Stage III (distant metastasis) (%)**	543	363 (15.5%)	180 (43.7%)	
**Histological classification**				
**Seminoma (*n*) (%)**	994 (36.1%)	860 (36.7%)	134 (32.5%)	<.001
**Non-seminoma (*n*) (%)**	1761 (63.9%)	1483 (63.3%)	278 (67.5%)	<.001
**Non-seminoma subtypes**				
**Pure teratoma (*n*) (%)**	93 (5.3%)	76 (5.1%)	17 (6.1%)	
**Choriocarcinoma (*n*) (%)**	18 (1.0%)	9 (0.6%)	9 (3.2%)	
**Embryonal carcinoma (*n*) (%)**	49 (2.8%)	45 (3.0%)	4 (1.4%)	
**Yolk sac tumor (*n*) (%)**	35 (2.0%)	29 (2.0%)	6 (2.2%)	
**Mixed (*n*) (%)**	1566 (88.9%)	1321 (89.1%)	245 (88.1%)	

Abbreviation: TGCT, testicular germ cell tumors.

Continuous variables are presented as medians with interquartile ranges defined as the 25th (Q1) to the 75th (Q3) percentiles (Q1-Q3), and categorical variables are presented as counts with percentages.

a Percentages for clinical stage are calculated based on the subset of patients with available clinical staging information, using the AJCC 8th edition classification.

The primary endpoint was 5-year OS, defined as time from diagnosis to death from any cause, with data censored at the last follow-up

### Skeletal muscle assessment

Skeletal muscle assessment was performed using the linear measurement method previously validated by Avrutin et al. and Steele et al.,^19^ utilizing Horos medical imaging software (Horos Project). Axial computed tomography slices were analyzed at the mid-L3 vertebral level, identified by the visualization of both transverse processes. Orthogonal linear measurements were performed bilaterally on the psoas and paraspinal (erector spinae and multifidus) muscles using digital calipers. For each muscle, the maximal transverse (width) and anterior-posterior (height) diameters were measured to form a perpendicular intersection. The cross-sectional area (CSA) for each muscle was estimated by multiplying these 2 diameters (Width × Height). The total estimated skeletal muscle area was calculated as the sum of the estimated areas of the right and left psoas and paraspinal muscles. Finally, the LMI was derived by normalizing the total muscle CSA to the patient’s height squared (cm^2^/m^2^).

To ensure methodological rigor, inter-observer reproducibility was assessed on a random subsample of 20% of the imaging cohort. The analysis yielded an intraclass correlation coefficient (ICC) of 0.98 (95% CI: 0.96-0.99), indicating excellent reliability for the linear measurement method. Due to the lack of established LMI cut-offs for the Mexican population, LMI was prioritized as a continuous variable or analyzed by cohort-specific distribution to avoid applying potentially invalid external standards.

### Statistical analysis

Data were summarized using medians and Q1 and Q3 for continuous variables, and frequencies with percentages for categorical variables. Group comparisons were performed using the Mann–Whitney *U* test or chi-square χ^2^ test, as appropriate. Prior to analysis, the inter-observer reliability of CT-based skeletal muscle measurements was validated on a random subsample (*n* = 46) using the ICC based on 2-way random-effects model (single measures) (Table S1).

#### Unsupervised classification and survival analysis

To identify integrated metabolic-nutritional patterns, we performed PCA followed by K-means clustering. The optimal number of clusters was determined using scree plots and variable contribution analysis. Survival outcomes among the identified metabolic profiles were estimated using the Kaplan–Meier method and compared using the log-rank test.

#### Association and prediction models

To assess factors associated with mortality, we utilized Cox proportional hazards regression models adjusted for clinical covariates. In the imaging sub-cohort, to evaluate the independent prognostic value of body composition while avoiding collinearity, we constructed 2 separate parsimonious multivariate models: one including BMI and another including LMI. Both models were adjusted for Age, IGCCCG Risk Group, Serum Albumin, and HDL (Table 2). Subsequently, to quantify the specific predictive performance of metabolic markers, we built multivariable logistic regression models for 5-year mortality in patients with seminoma and non-seminoma (Table S2). Two modeling strategies were compared: a complete model (including age, BMI, LMI, albumin, and full lipid profile) and a reduced model (BMI, LMI, and albumin). Model discrimination was assessed using the area under the curve (AUC).

**Table 2.** Univariate and multivariate analysis of clinical, nutritional, and metabolic factors associated with mortality in patients with TGCT.

Variables	Logistic regression		Cox regression		Univariate analysis		
	OR (95% CI)	*P*-value	HR (95% CI)	*P*-value	Alive patients	Deceased patients	*P*-value
**Metabolic sub-cohort (*n* = 586)**							
**Age (years)**	1.06 (1.02, 1.10)	.007	1.05 (1.03, 1.07)	<.001	28.4	31.6	.007
**BMI (kg/m^2^)**	0.95 (0.84, 0.97)	.05	0.91 (0.86, 0.97)	.006	25.11	23.73	<.001
**Stage**	2.04 (1.34, 3.19)	<.001	1.40 (1.11, 1.76)	<.001	1	2-3	<.001
**Albumin (g/dL)**	0.08 (0.03, 0.17)	<.001	0.29 (0.20, 0.41)	<.001	4.11	3.17	<.001
**Glucose (mg/dL)**	1.00 (0.98, 1.03)	.80	1.00 (0.99, 1.02)	.52	94.16	95.93	.28
**Triglycerides (mg/dL)**	0.98 (0.97, 0.99)	<.001	0.99 (0.99, 0.99)	.005	205.77	111.73	<.001
**Total cholesterol (mg/dL)**	0.96 (0.94, 0.98)	<.001	0.99 (0.98, 1.00)	.023	182.42	134.37	<.001
**HDL (mg/dL)**	0.85 (0.79, 0.89)	<.001	0.95 (0.93, 0.97)	<.001	40.42	31.62	<.001
**LDL (mg/dL)**	0.97 (0.97, 0.98)	.05	1.00 (0.99, 1.01)	.57	115.02	92.16	<.001
**Imaging sub-cohort (*n* = 231) BMI (kg/m^2^) analysis**							
**Age (years)**	1.05 (0.99-1.11)	.08	1.03 (1.00-1.05)	.027			
**BMI/LMI**	0.81 (0.67-0.97)	.034	0.92 (0.84-0.99)	.047			
**Risk group (IGCCCG)**	5.24 (2.85-10.6)	<.001	2.11 (1.50-2.95)	<.001			
**Albumin (g/dL)**	0.03 (0.01-0.09)	<.001	0.23 (0.15-0.36)	<.001			
**HDL (mg/dL)**	0.84 (0.78-0.90)	<.001	0.94 (0.91-0.96)	<.001			
**Imaging sub-cohort (*n* = 231) LMI (kg/m^2^) analysis**							
**Age (years)**	1.04 (0.98-1.10)	.152	1.02 (0.99-1.04)	.145			
**BMI/LMI**	0.89 (0.80-0.98)	.026	0.95 (0.90-0.99)	.047			
**Risk group (IGCCCG)**	4.86 (2.65-9.74)	<.001	2.09 (1.48-2.94)	<.001			
**Albumin (g/dL)**	0.03 (0.01-0.10)	<.001	0.27 (0.18-0.42)	<.001			
**HDL (mg/dL)**	0.84 (0.77-0.90)	<.001	0.94 (0.91-0.96)	<.001			

Abbreviations: BMI, body mass index; HDL, high-density lipoprotein; HR, hazard ratio; IGCCCG, International Germ Cell Cancer Collaborative Group; LMI, lean mass index; OR, odds ratio.

The metabolic sub-cohort (*n* = 586) section displays baseline characteristics stratified by vital status and univariate regression analysis for the full cohort. The Imaging sub-cohort (*n* = 231) section presents multivariate logistic and Cox regression analyses. Two separate models were constructed to evaluate body composition: one including BMI and one including LMI. Both multivariate models are adjusted for Age, HDL, IGCCCG risk group, and serum albumin.

#### Model diagnostics and validation

To address potential collinearity between anthropometric variables (BMI and LMI), a variance inflation factor (VIF) analysis was performed (Table S3). Finally, internal validation of the predictive models was conducted to correct optimism using a bootstrapping technique with 2000 resamples. All analyses were performed using R software (version v4.3.0), and a *P*-value < .05 was considered statistically significant.

### Ethical considerations

The study was approved by the INCan Research and Ethics Committees (012/031/ICI, CEI/783/12, CEI/052/22) and conducted following the Declaration of Helsinki, with a waiver of informed consent.

## Results

### Clinical and demographic characteristics

Our analysis included 2755 patients with confirmed TGCT, from which a metabolic sub-cohort (*n* = 586) and an imaging sub-cohort (*n* = 231) were established. Both sub-cohorts were representative of the full cohort (Figure S1 [see online supplementary material for a color version of this figure] and Table S4).

**Figure 1.**
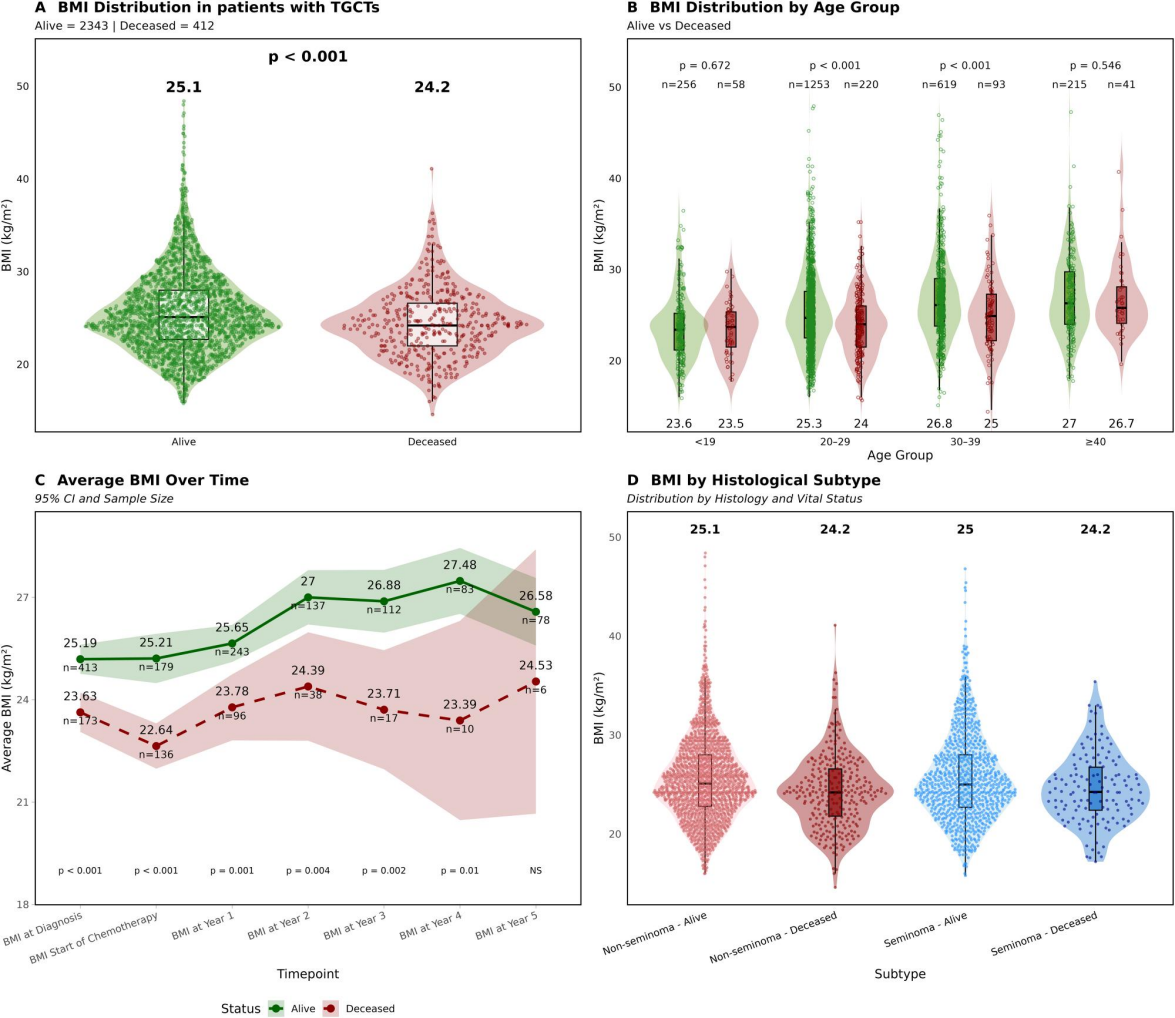
Differences in BMI by survival status, age, histology, and over time in patients with TGCT. (A) BMI distribution at diagnosis according to vital status. At diagnosis, patients who died exhibited significantly lower BMI values compared to survivors, highlighting early differences in nutritional status. (B) BMI stratified by age group and vital status. BMI remained lower among patients who died across all age groups, with the largest differences observed in younger individuals. (C) Longitudinal BMI trajectories from diagnosis through follow-up. Longitudinal follow-up revealed a persistent gap in BMI between survivors and patients who died, with divergence starting at diagnosis and widening over time. (D) Comparison of BMI between patients with seminomatous and non-seminomatous tumors, stratified by vital status. Across both seminomatous and non-seminomatous tumors, patients who died exhibited significantly lower BMI compared to alive.

Baseline characteristics differed significantly between patients who survived and those who died. Patients who died were slightly younger, had a lower BMI, a higher prevalence of recreational drug use (Table S5), and presented more frequently with advanced-stage (Stage III) disease. Histologically, non-seminomatous tumors, particularly the choriocarcinoma subtype, were also more frequent in the group of patients who died (Table 1). A lower BMI at diagnosis was a consistent finding among these patients (Figure 1A), a disparity that was most pronounced in the 20-39-year age range (Figure 1B).

Longitudinal analysis showed that the lower BMI observed in patients who died was a gap that persisted throughout the course of treatment (Figure 1C). Patients with non-seminoma exhibited significantly lower BMI values than those with seminoma. Among patients who died within the non-seminoma group, the lowest BMI values were recorded in the mixed subtype (Figure 1D, Figure S2 [see online supplementary material for a color version of this figure]).

**Figure 2.**
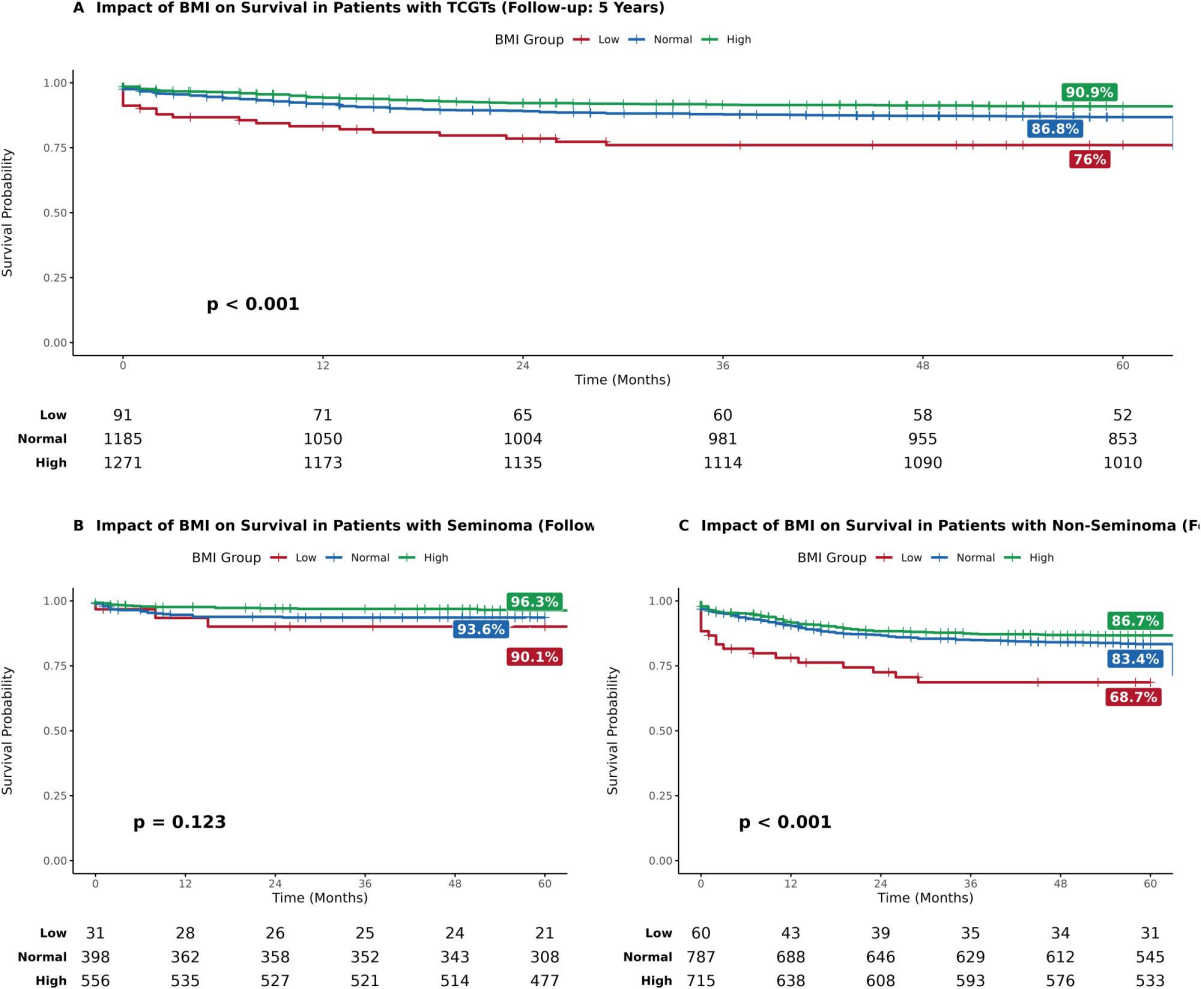
Kaplan–Meier survival curves by BMI group in patients with TGCT. (A) Overall, 5-year survival in TGCT patients according to BMI category (low BMI, <18.5; normal BMI, 18.5-24.9; and high BMI, >25). Five-year survival in patients with low BMI was 76.0%, compared with 86.8% in patients with normal BMI and 90.9% in patients with high BMI. (B) Overall, 5-year survival in seminomatous tumors. Five-year survival in patients with seminoma with low BMI was 90.1%, compared with 93.6% in patients with normal BMI and 96.3% in patients with high BMI. (C) Overall, 5-year survival in patients with non-seminoma. Five-year survival in patients with non-seminoma with low BMI was 68.7%, compared with 83.4% in patients with normal BMI and 86.7% in patients with high BMI.

Consequently, BMI at diagnosis was significantly associated with 5-year survival. In the entire cohort, patients with low BMI (<18.5 kg/m^2^) had a 5-year survival rate of only 76.0%, significantly lower than the 90.9% observed in patients with high BMI (*P* < .0001) (Figure 2A).

This effect was particularly evident among patients with non-seminoma, where low BMI was associated with a 5-year survival of just 68.7%, compared to 86.7% for those with high BMI in the same group (*P* < .001) (Figure 2C). In contrast, survival outcomes for patients with seminoma were more favorable across all BMI categories (Figure 2B).

**Figure 3.**
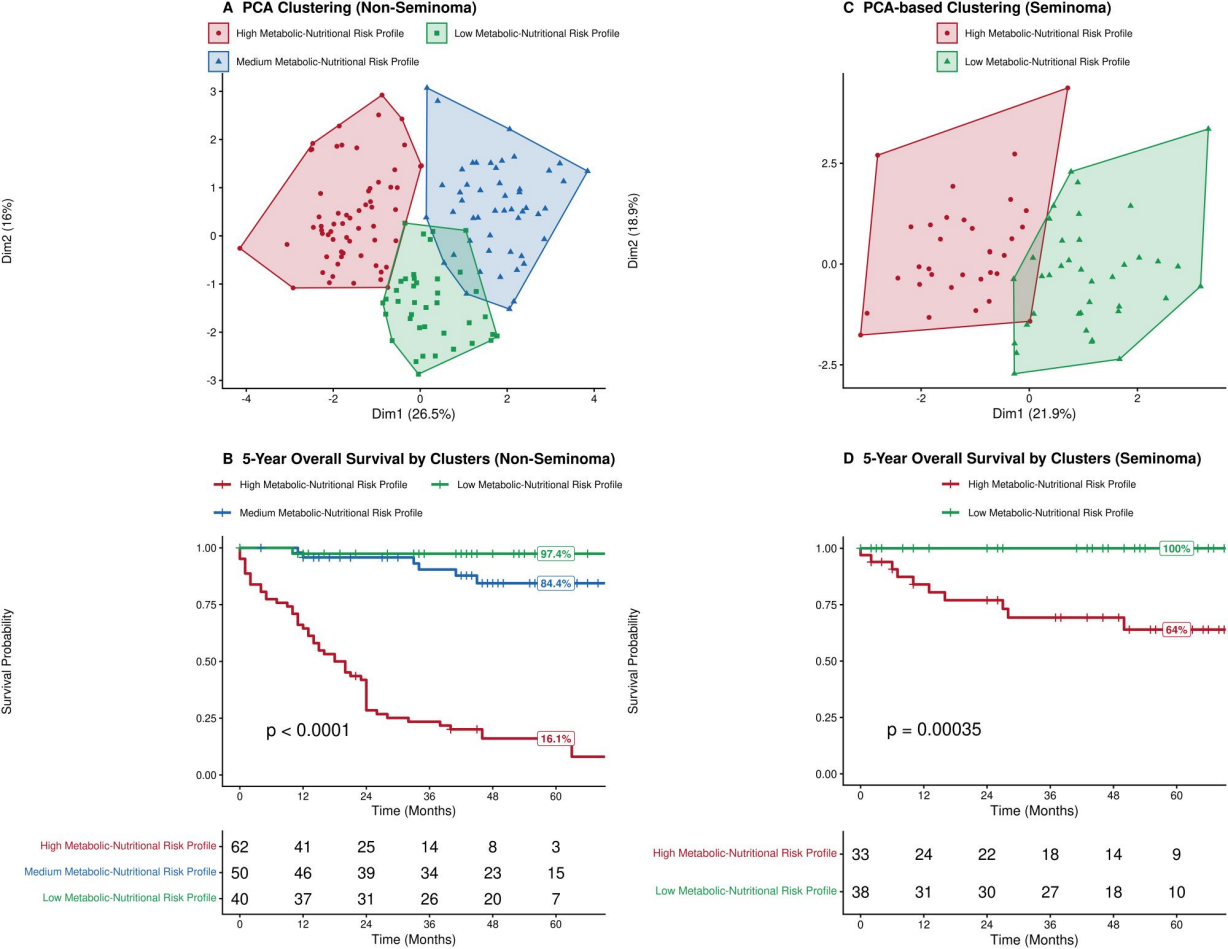
Metabolic-nutritional risk profiles and survival outcomes in the full study cohort (all IGCCCG risk groups). (A) PCA-based clustering in patients with non-seminoma revealed 3 distinct profiles: a high-risk profile (red) characterized by severe depletion, medium-risk profile (blue) with intermediate features, and a low-risk profile (green) with preserved metabolic reserves. (B) Kaplan–Meier curves show marked OS differences: 5-year OS was 16.1% in the high-risk group, followed by 84.4% in low-risk 1 group and 97.4% in low-risk 2 group (*P *< .0001), supporting the prognostic value of metabolic clustering in patients with non-seminoma. (C) In patients with seminoma, PCA clustering identified 2 distinct groups: a high-risk cluster (red) with lower metabolic reserves and a low-risk cluster (green) with robust status. (D) Kaplan–Meier curves show marked OS differences: 5-year OS was 64.0% in the high-risk group, followed by 100% in low-risk group (*P *< .0001), highlighting the prognostic relevance of metabolic profiling in across histological subtypes.

Evaluation of baseline metabolic parameters in the metabolic sub-cohort (*n* = 586) revealed a significantly depleted profile in patients who died. Univariate analysis showed that these patients exhibited lower concentrations of serum albumin, total cholesterol, HDL, and triglycerides (*P* < .001 for all comparisons; Table 2, Table S6, and Figure 3). The median albumin in patients who died was 3.17 g/dL.

To evaluate the specific impact of skeletal muscle mass, we analyzed the imaging sub-cohort (*n* = 231), comprising patients with available baseline abdominal CT scans suitable for morphometric analysis. Inter-observer reproducibility was excellent, with an ICC of 0.98 (95% CI: 0.96-0.99), indicating excellent reliability for the linear measurement method. In this specific group, patients who died consistently exhibited significantly lower LMI values compared to survivors.

This muscle depletion was evident across all age strata, with differences often exceeding 3.5 units between groups (Table S7). While this disparity was significant across all ages, it was most pronounced in the primary TGCT age range of 20-29 years (*P* < .0001). These univariate findings highlighted an initial association between muscle mass depletion and adverse outcomes, particularly in younger individuals, warranting further adjustment for tumor burden.

In the initial multivariate Cox proportional hazards analysis including all covariates, LMI did not reach statistical significance (HR 0.99; 95% CI 0.94-1.04, *P* = .607), while IGCCCG risk and albumin remained significant (Table S7). Two separate parsimonious models were subsequently constructed: one incorporating BMI and another incorporating LMI (Table 2). VIF analysis showed all values were < 2.0.

To resolve this collinearity and accurately evaluate the independent prognostic value of body composition, we subsequently constructed 2 separate parsimonious models: one incorporating BMI and another incorporating LMI (Table 2). VIF analysis confirmed the absence of multicollinearity in these optimized models (all VIF values < 2.0).

In the LMI model adjusted for age, IGCCCG risk, serum albumin, and HDL, skeletal muscle mass retained statistical significance as an independent prognostic factor (HR 0.95 95% CI 0.90-0.99; *P* = .047). Similarly, in the BMI model, total body mass was also independently associated with survival (HR 0.92; 95% CI 0.84-0.99, *P* = .047). Across both models, the IGCCCG risk classification remained the dominant factor associated with mortality, and serum albumin maintained significant independent association with survival (HR 0.27; 95% CI 0.18-0.42, *P* < .001).

To identify underlying clinical-nutritional patterns, we performed PCA and clustering on patients with non-seminoma. The first principal component (PC1) represented a clear axis of metabolic reserve, driven primarily by BMI, LMI, albumin, and lipid levels (Figures S3 and S4, see online supplementary material for a color version of these figures). Based on this, unsupervised K-means clustering identified 3 distinct patient metabolic-nutritional risk profiles (Table 3, Figure 3A). These metabolically defined clusters were associated with distinct OS outcomes (Figure 3B).

**Table 3.** Baseline clinical and metabolic-nutritional characteristics of patients with TGCT stratified by histology and PCA-clusters (all IGCCCG risk groups).

Markers/Parameters	**Non-**s**eminoma^a^**			Seminoma^b^	
	High metabolic-nutritional risk profile (*N* = 62) 5y OS = 16.1%	Medium metabolic-nutritional risk profile (*N* = 50) 5y OS = 84.4%	Low metabolic-nutritional risk profile (*N* = 40) 5y OS = 97.4%	High metabolic-nutritional risk profile (*N* = 33) 5y OS= 64%	Low metabolic-nutritional risk profile (*N* = 38) 5y OS= 100%
**BMI (kg/m²)**	23.12	26.83	24.47	23.33	26.48
**LMI**	22.07	29.89	28.09	23.1	30.75
**IGCCCG risk groups**	Intermediate-Poor	Good-Intermediate	Good-Intermediate	Good-Intermediate	Good-Intermediate
**Albumin (g/dL)**	3.36	4.14	4.54	3.81	4.49
**Glucose (mg/dL)**	93.67	92.78	89.78	89.79	90.71
**Total cholesterol (mg/dL)**	146.97	213.14	144.05	173.97	188.97
**HDL (mg/dL)**	34.12	39.1	37.9	45.91	38.09
**LDL (mg/dL)**	101.07	133.81	90.76	106.13	113.53
**Triglycerides (mg/dL)**	123.97	290.01	168.66	129.67	209.26
**Age (years)**	25.77	30.18	24.18	23.33	26.48

a Patients with non-seminoma: The High Metabolic-Nutritional Risk Profile (Cluster 1) exhibited the most depleted clinical status, distinguished by the lowest albumin and LMI, alongside elevated glucose levels suggestive of metabolic stress. The Medium Metabolic-Nutritional Risk Profile (Cluster 2) showed intermediate nutritional markers, though lipid parameters remained comparable to the high-risk group. The Low Metabolic-Nutritional Risk Profile (Cluster 3) demonstrated the most favorable phenotype, characterized by the highest albumin levels, robust lipid reserves, and preserved muscle mass.

b Patients with seminoma: The High Metabolic-Nutritional Risk Profile (Cluster 1) was characterized by significantly lower BMI, LMI (sarcopenia), serum albumin, and lipid levels (including total cholesterol, LDL, and triglycerides). In contrast, the Low Metabolic-Nutritional Risk Profile (Cluster 2) displayed a preserved morpho-metabolic status with higher muscle mass and lipid reserves.

IGCCCG Classification: This analysis includes all IGCCCG prognostic groups available for each histology.

This prognostic separation persisted even when analyzing only patients with advanced disease. In a sub-analysis restricted to IGCCCG intermediate and poor-risk patients with non-seminoma, showed a 5-year survival of 14.9% for those with a depleted profile, compared to 68.1% for those with a preserved metabolic profile (Table S8).

A similar analysis in patients with advanced-stage seminoma revealed that PC1 again represented a cohesive axis of metabolic reserve, driven by BMI, LMI, albumin, and lipids (Figure S5, see online supplementary material for a color version of this figure). K-means clustering based on this component identified 2 distinct patient groups (Table 3, Figure 3C). In terms of survival, the metabolically depleted group had a rate of 64.0%, compared to 100% in the low-risk group (Figure 3D). We also attempted a sensitivity analysis restricted to the intermediate-risk seminoma subgroup; however, due to the limited sample size (*n* = 9), definitive clustering conclusions could not be drawn (Table S8).

**Figure 4.**
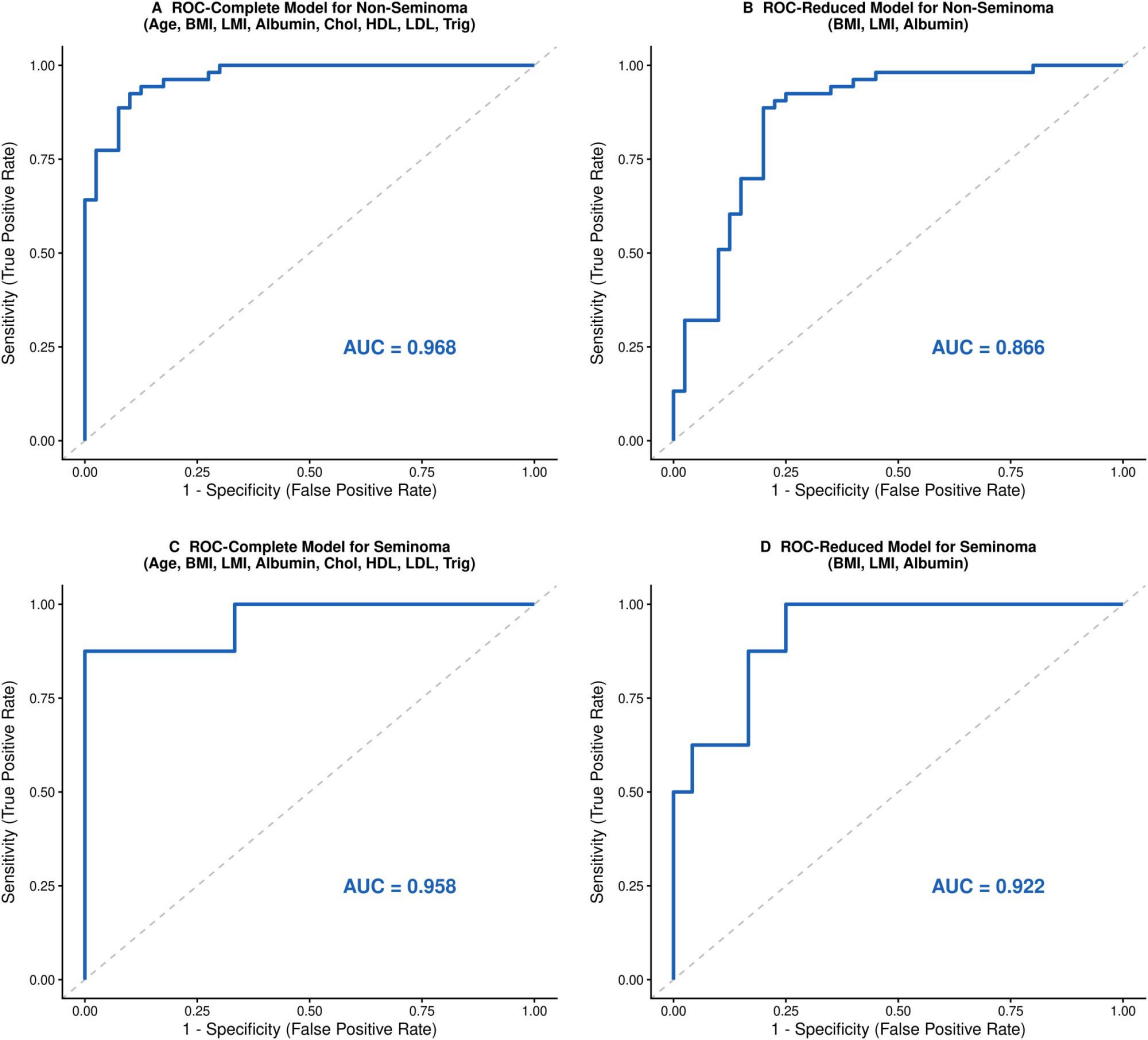
ROC curves for metabolic models predicting mortality in TGCT. (A) The Complete model (including age, BMI, LMI, albumin, total cholesterol, HDL, LDL, and triglycerides) in patients with non-seminoma demonstrated high discriminative ability with a bootstrap-validated AUC of 0.968. (B) The Reduced model (incorporating only BMI, LMI, and albumin) in patients with non-seminoma maintained robust performance with an optimism-corrected AUC of 0.866, balancing prognostic accuracy with parsimony. (C) The Complete model in patients with seminoma achieved a validated AUC of 0.958. (D) The Reduced model in patients with seminoma showed a validated AUC of 0.922. AUC values represent optimism-corrected metrics derived from internal validation using bootstrapping (2000 iterations).

Finally, we evaluated the predictive utility of these markers using ROC curve analysis and internal validation via bootstrapping (2000 iterations) (Figure 4).

In patients with non-seminoma, the complete model achieved an apparent AUC of 0.968 (Figure 4A); however, acknowledging the potential for overfitting due to high dimensionality, we prioritized the reduced model (incorporating BMI, LMI, and albumin). This parsimonious model demonstrated a stable bootstrap-validated AUC of 0.866 (95% CI: 0.781-0.938) (Figure 4B). This confirms that this parsimonious set of 3 markers maintains its association with mortality risk, achieving a sensitivity of 88.7%, and a specificity of 80.0% (Table S2).

For patients with seminoma, the models demonstrated discriminative potential, with the reduced model achieving a validated AUC of 0.922 (95% CI: 0.812-0.990) (Figure 4C and D). However, these associations should be interpreted with caution given the lower number of mortality events in the seminoma subgroup, which is reflected in the wider confidence intervals compared to the non-seminoma cohort (Table S2).

## Discussion

Our findings support the prognostic relevance of host metabolic status in TGCT particularly in a Mexican cohort where this dimension has been underexplored. We observed that a lower BMI was associated with higher mortality, a trend that holds true for both seminoma and non-seminoma subtypes. This lower BMI at diagnosis was a consistent finding among patients who died, creating a gap that persisted throughout the course of treatment. This difference was closely linked to histology; specifically, the lower BMI observed in the non-seminoma group was driven primarily by the mixed subtype. This result aligns with the “obesity paradox” seen in other malignancies.^13^ This paradox suggests that a higher BMI may not only reflect greater metabolic reserve but also confer a survival advantage, potentially by allowing patients to better tolerate treatment toxicity.^13^^,^^15^^,^^20^^,^^21^ In our cohort, this finding was particularly relevant for patients with non-seminoma, a histology known for its more aggressive behavior. Interestingly, these patients were younger and had a lower BMI at diagnosis. This pattern suggests that the low BMI in this subgroup may not simply reflect lifestyle factors, rather, it may be a manifestation of intrinsic metabolic derangements associated with the dynamic host–tumor interaction. However, while BMI serves as an accessible proxy, its clinical utility is limited by its inability to distinguish lean mass from adipose tissue. Consequently, future research should prioritize exploring body composition using more sensitive methods, such as bioelectrical impedance analysis (BIA) or dual-energy X-ray absorptiometry (DXA), to better characterize critical clinical phenotypes, including sarcopenia and sarcopenic obesity—that may modulate treatment tolerance and outcomes

A central finding of this study is the independent association between serum albumin and overall survival (HR 0.27; 95% CI: 0.18-0.42; *P* < .001), retaining its significance even after adjustment for the standard IGCCCG risk classification. Our results are consistent with findings in other malignancies where hypoalbuminemia is a well-established marker of poor prognosis.^10^^,^^14^ In our cohort, the median albumin in patients who died (3.17 g/dL) was indicative of clinical hypoalbuminemia. In the context of advanced cancer is a potent proxy for systemic inflammation and high cytokine activity (eg, IL-6, TNF-alpha), which promotes catabolism and suppresses albumin synthesis.^10^^,^^14^^,^^22^

This independent prognostic association suggests that the host’s inflammatory response plays a critical role in survival outcomes, providing information that complements tumor-centric variables. However, as direct inflammatory mediators were not assessed in this study, future research should validate these findings by integrating specific markers of systemic inflammation to confirm this mechanism.

Similarly, our study revealed a consistent pattern of lipid depletion, particularly HDL showed an independent association with higher mortality (HR 0.94; 95% CI: 0.91-0.96; *P* < .001). This finding contrasts with what has been observed in other types of cancer, where elevated lipid levels can be indicative of an unfavorable prognosis.^23^^,^^24^ While this phenomenon could hypothetically reflect a hypermetabolic state or tumor consumption,^25^ the exact mechanisms driving this paradoxical depletion in TGCT remain to be fully elucidated. It raises critical questions regarding whether this stems from tumor biology, systemic malnutrition, or altered dietary patterns. Therefore, rather than implying immediate causality, these findings serve as a precedent for future research dedicated to investigating nutritional status and macronutrient intake, laying the groundwork for targeted clinical nutrition projects in this population.

Taken together, hypoalbuminemia and lipid depletion may suggest deeper systemic metabolic stress, perhaps involving inflammation and endothelial dysfunction. As these markers are detectable at the time of diagnosis, they serve as early warning signals for potentially adverse evolution. Identifying these vulnerable patients allows for a more comprehensive strategy, prompting early referral to multidisciplinary support services—such as clinical nutrition—to optimize patient care. While this retrospective analysis generates the hypothesis that metabolic depletion is associated with poorer survival, prospective validation is required to determine if correcting these deficits actively improves outcomes. Ultimately, this perspective complements standard tumor-centric staging by adding a layer of host-related metabolic granularity to the prognostic assessment

Assessing skeletal muscle mass via LMI provided prognostic information complementary to BMI, allowing for the differentiation of lean tissue from adiposity—a distinction BMI cannot make.^13^ Our analysis identified LMI as an independent factor associated with overall survival (HR 0.95; 95% CI: 0.90-0.99; *P* = .047) after adjusting for tumor burden. This finding suggests that skeletal muscle depletion is associated with increased mortality risk, aligning with previous reports linking low muscle mass to increased susceptibility to treatment-related toxicity and adverse outcomes.^26^^,^^27^ Consequently, the independent significance of both BMI (*P* = .047) and LMI reinforces the potential utility of assessing body composition. This perspective complements standard staging by helping to identify specific vulnerable phenotypes that may be at risk for adverse outcomes, suggesting a need for closer clinical attention in this subset of patients.

PCA-based clustering allowed for the identification of a clearly defined metabolic-nutritional risk profile. This patient cluster, characterized by low LMI, hypoalbuminemia, and lipid depletion, presented features consistent with metabolic depletion^28^ and exhibited a markedly poorer survival. This suggests that the survival benefit associated with the “obesity paradox” may largely reflect a state of preserved metabolic reserves. In this context, a higher BMI likely serves as a proxy for adequate muscle mass and nutritional status acting as a metabolic buffer, rather than adiposity alone conferring the protective effect.^18^^,^^29^

To address the limitations inherent to retrospective modeling, g and the high dimensionality of our data, specifically the collinearity, between anthropometric and metabolic markers—we prioritized parsimonious models and performed internal validation using bootstrapping. The optimism-corrected AUC of 0.866 for our reduced model (BMI, LMI, and Albumin) suggests that these accessible markers provide stable prognostic information. However, rather than serving as a replacement for precision diagnostics, this parsimonious approach serves as a pragmatic first step for integrating “metabolic-aware” assessments into routine oncology practice. Ultimately, these findings highlight the potential value incorporating specialized Clinical Nutrition services and aspiring toward the routine implementation of more objective body composition technologies—such as bioelectrical impedance analysis—to align with international standards of comprehensive cancer care.

Despite the dominance of tumor burden variables, our exploratory metabolic-nutritional risk profiles demonstrated potential clinical utility by capturing prognostic variance that standard staging may miss. This was most evident in the sub-analysis of patients classified as “Poor Risk” by IGCCCG (Table S8). Within this theoretically homogeneous high-risk group, our metabolic profiling distinguished 2 divergent trajectories: patients with a preserved metabolic profile achieved a 5-year survival of 68.1%, whereas those with a depleted profile had a survival of only 14.9%. These findings suggest a potential rationale for the future integration of metabolic assessments into a more comprehensive clinical approach. Rather than viewing high-risk patients as a uniform group, metabolic profiling may help identify vulnerable individuals who could benefit from personalized nutritional support, though prospective validation is required to confirm the impact on survival outcomes.

In summary, our study proposes an integrated risk approach combining metabolic profiling and body composition in TGCT. By characterizing a high-risk metabolic-nutritional profile—defined by low BMI, low LMI, hypoalbuminemia, and an altered lipid profile—we highlight a vulnerability axis that provides prognostic information complementary to conventional tools. However, to translate these retrospective associations into clinical practice, the immediate priority is prospective validation. Future research must focus on confirming the prognostic weight of these markers in controlled settings to establish their definitive utility. In parallel, efforts should aim to elucidate the specific molecular pathways linking these metabolic signatures to tumor biology, potentially drawing upon established nutritional paradigms in other malignancies to fully understand the host–tumor interaction in TGCT.

Our study has several limitations inherent to its design. First, as a retrospective, single-center analysis, the findings are subject to selection bias and may not be fully generalizable to populations with different ethnic or socioeconomic backgrounds.^8^ Second, although we performed internal validation using bootstrapping to correct optimism and demonstrate model stability, the lack of an external validation cohort limits the immediate clinical applicability of the proposed prognostic model. Third, regarding body composition assessment, while CT-derived LMI is validated and accessible proxy,^19^ it primarily measures muscle quantity without capturing muscle quality or functionality. A definitive diagnosis of sarcopenia requires assessing both reduced muscle mass and diminished strength (eg, via handgrip dynamometry), metrics that were not available in this retrospective cohort. Furthermore, we did not employ full body composition techniques—such as BIA or DXA, which would offer a more precise determination of adipose and lean tissue distribution.

Finally, we lacked key inflammatory biomarkers (such as C-reactive protein) and could not account for dynamic changes in nutritional status during chemotherapy, preventing us from assessing the evolution of physiological reserve over time.^30^ This limitation points to the potential need for closer clinical nutritional surveillance in routine practice. Consequently, future multicenter prospective studies are warranted not only to confirm these associations but to determine if integrating rigorous nutritional evaluation and follow-up into the standard clinical approach actively translates into maximized survival opportunities for these patients.

## Conclusions

Host metabolic status at diagnosis—specifically characterized by severe hypoalbuminemia, skeletal muscle depletion and an altered lipid profile—are associated with mortality in patients with TGCT. LMI and serum albumin were identified as independently associated factors, providing information complementary to the IGCCCG classification. Furthermore, our exploratory metabolic-nutritional risk profiles effectively stratify survival outcomes, identifying high-risk patients with metabolic vulnerability who may benefit from early supportive interventions. These findings support the integration of metabolic and body composition profiling into clinical research and practice warranting future prospective validation to confirm their clinical utility.

## Supplementary Material

oyag072_Supplementary_Data

## Data Availability

The data that support the findings of this study are available on reasonable request from the corresponding author (R.G.-B.: rodrigop@ciencias.unam.mx)
