# Supplementary material for: Metabolic profile and skeletal muscle as predictors of survival in testicular germ cell tumors
Source: Oncologist. 2026 Apr 16;31(5):oyag072. doi: 10.1093/oncolo/oyag072 (PMC13092131; doi:10.1093/oncolo/oyag072)
Supplement: oyag072_Supplementary_Data [file oyag072_supplementary_data.zip › renamed_39ae4.docx]

**Supplementary Table 3. Assessment of Multicollinearity Among Clinical and Metabolic Covariates Using Variance Inflation Factor (VIF) Stratified by Histology.**

| **Variable** | **Imaging**  **sub-cohort (VIF)** | **Patients with**  **Non-Seminoma (VIF)** | **Patients**  **with Seminoma (VIF)** |
| --- | --- | --- | --- |
| Age | 1.25 | 1.19 | 1.71 |
| BMI (kg/m²) | 1.39 | 1.45 | 1.91 |
| LMI (kg/m²) | 1.48 | 1.54 | 1.5 |
| IGCCCG | 1.65 | 1.75 | 1.48 |
| Albumin | 1.54 | 1.63 | 1.53 |
| HDL Cholesterol | 1.18 | 1.18 | 1.28 |
| Total Cholesterol | 1.43 | 1.47 | 1.45 |
| Triglycerides | 1.25 | 1.25 | 1.72 |

**Note**: VIF values quantify the severity of multicollinearity in regression analysis. A VIF < 5 is generally considered evidence of no significant multicollinearity. In this cohort, all VIF values were < 2.0, confirming that the selected predictors (Age, BMI, LMI, Albumin, and Lipids) provide independent information and are suitable for inclusion in the multivariable models. **Abbreviations:** BMI, Body Mass Index; LMI, Lean Mass Index; IGCCCG, International Germ Cell Cancer Collaborative Group; HDL, High-Density Lipoprotein; VIF, Variance Inflation Factor.
