# Supplementary material for: Metabolic profile and skeletal muscle as predictors of survival in testicular germ cell tumors
Source: Oncologist. 2026 Apr 16;31(5):oyag072. doi: 10.1093/oncolo/oyag072 (PMC13092131; doi:10.1093/oncolo/oyag072)
Supplement: oyag072_Supplementary_Data [file oyag072_supplementary_data.zip › renamed_69b99.docx]

**Supplementary Table 5.** Association between substance use and mortality in patients with TGCTs.

| **Variables** | **Univariate Analysis** | | | **Logistic Regression** | | **Cox Regression** | |
| --- | --- | --- | --- | --- | --- | --- | --- |
|  | **Alive Patients** | **Deceased Patients** | **p-value** | **OR (95%CI)** | **p-value** | **HR (95%CI)** | **p-value** |
| Substance Use | 300 | 87 | 0.485 | 0.84 (0.61, 1.27) | 0.422 | 0.91 (0.62,1.32) | 0.615 |
| Smoking | 322 | 91 | 0.7082 | 0.90 (0.66, 1.45) | 0.628 | 0.98 (0.66,1.45) | 0.914 |
| Alcohol Use | 306 | 87 | 0.669 | 0.89 (0.65, 1.39) | 0.593 | 0.95 (0.65, 1.39) | 0.805 |
| Drug Use | 443 | 118 | 0.2905 | 1.77 (0.94, 3.96) | 0.197 | 1.93 (0.94, 3.96) | 0.072 |

Table summarizes the univariate, logistic regression, and Cox proportional hazards analyses for substance use (including all variables) and independent variables such as smoking, alcohol and illicit drug use (including marijuana). No statistically significant associations were found with mortality in this cohort.
