# Supplementary material for: Metabolic profile and skeletal muscle as predictors of survival in testicular germ cell tumors
Source: Oncologist. 2026 Apr 16;31(5):oyag072. doi: 10.1093/oncolo/oyag072 (PMC13092131; doi:10.1093/oncolo/oyag072)
Supplement: oyag072_Supplementary_Data [file oyag072_supplementary_data.zip › renamed_601c1.docx]

**Table 3.** Baseline Clinical and Metabolic-Nutritional Characteristics of Patients with TGCT Stratified by Histology and PCA-Clusters (All IGCCCG Risk Groups).

| **Markers/ Parameters** | **Non-Seminoma^a^** | | | **Seminoma^b^** |  |
| --- | --- | --- | --- | --- | --- |
|  | **High Metabolic-Nutritional Risk Profile (N=62) 5y OS= 16.1%** | **Medium Metabolic-Nutritional Risk Profile (N=50) 5y OS= 84.4%** | **Low Metabolic-Nutritional Risk Profile (N=40) 5y OS= 97.4%** | **High Metabolic-Nutritional Risk Profile (N=33) 5y OS= 64%** | **Low Metabolic-Nutritional Risk Profile (N=38) 5y OS= 100%** |
| **BMI (kg/m²)** | 23.12 | 26.83 | 24.47 | 23.33 | 26.48 |
| **LMI** | 22.07 | 29.89 | 28.09 | 23.1 | 30.75 |
| **IGCCCG Risk Groups** | Intermediate-Poor | Good-Intermediate | Good-Intermediate | Good- Intermediate | Good- Intermediate |
| **Albumin (g/dL)** | 3.36 | 4.14 | 4.54 | 3.81 | 4.49 |
| **Glucose (mg/dL)** | 93.67 | 92.78 | 89.78 | 89.79 | 90.71 |
| **Total Cholesterol (mg/dL)** | 146.97 | 213.14 | 144.05 | 173.97 | 188.97 |
| **HDL (mg/dL)** | 34.12 | 39.1 | 37.9 | 45.91 | 38.09 |
| **LDL (mg/dL)** | 101.07 | 133.81 | 90.76 | 106.13 | 113.53 |
| **Triglycerides (mg/dL)** | 123.97 | 290.01 | 168.66 | 129.67 | 209.26 |
| **Age (years)** | 25.77 | 30.18 | 24.18 | 23.33 | 26.48 |

**^a^Patients with Non-Seminoma:** The High Metabolic-Nutritional Risk Profile (Cluster 1) exhibited the most depleted clinical status, distinguished by the lowest albumin and LMI, alongside elevated glucose levels suggestive of metabolic stress. The Medium Metabolic-Nutritional Risk Profile (Cluster 2) showed intermediate nutritional markers, though lipid parameters remained comparable to the high-risk group. The Low Metabolic-Nutritional Risk Profile (Cluster 3) demonstrated the most favorable phenotype, characterized by the highest albumin levels, robust lipid reserves, and preserved muscle mass.

**^b^Patients with Seminoma:** The High Metabolic-Nutritional Risk Profile (Cluster 1) was characterized by significantly lower BMI, LMI (sarcopenia), serum albumin, and lipid levels (including total cholesterol, LDL, and triglycerides). In contrast, the Low Metabolic-Nutritional Risk Profile (Cluster 2) displayed a preserved morpho-metabolic status with higher muscle mass and lipid reserves.

**IGCCCG Classification:** This analysis includes all IGCCCG prognostic groups available for each histology
