# Supplementary material for: Metabolic profile and skeletal muscle as predictors of survival in testicular germ cell tumors
Source: Oncologist. 2026 Apr 16;31(5):oyag072. doi: 10.1093/oncolo/oyag072 (PMC13092131; doi:10.1093/oncolo/oyag072)
Supplement: oyag072_Supplementary_Data [file oyag072_supplementary_data.zip › renamed_979ca.docx]

**Supplementary Table 1. Inter-observer Reproducibility Analysis of CT-based Skeletal Muscle Measurements.**

| **Parameter** | **n (Subset)** | **Statistical Model** | **Type** | **ICC Value** | **95% CI** | **P-value** |
| --- | --- | --- | --- | --- | --- | --- |
| **Lean Mass Index (LMI)** | 46 | Two-way random effects | Absolute Agreement  (Single Measures) | 0.98 | 0.96 – 0.99 | < 0.001 |
|  | 46 | Two-way mixed effects | Absolute Agreement  (Single Measures) | 0.98 | 0.96 – 0.99 | < 0.001 |

**Note**: Inter-observer reliability was assessed on a random subsample representing 20% of the total cohort (n=46). Calculations were performed using a two-way mixed-effects and random-effect model based on absolute agreement (Single Measures). Skeletal muscle assessment relied on the linear measurement method (orthogonal diameters), a validated surrogate for total muscle mass. An ICC value > 0.90 is interpreted as excellent reliability. **Abbreviations**: CI, Confidence Interval; ICC, Intraclass Correlation Coefficient; LMI, Lean Mass Index.
