# Supplementary material for: Metabolic profile and skeletal muscle as predictors of survival in testicular germ cell tumors
Source: Oncologist. 2026 Apr 16;31(5):oyag072. doi: 10.1093/oncolo/oyag072 (PMC13092131; doi:10.1093/oncolo/oyag072)
Supplement: oyag072_Supplementary_Data [file oyag072_supplementary_data.zip › renamed_a8c23.docx]

**Supplementary Table 6**. Comprehensive univariate and multivariate analysis of clinical, nutritional, and metabolic factors associated with mortality in the imaging sub-cohort (n=231).

| **Variables** | **Univariate Analysis** | | | **Logistic Regression** | | **Cox Regression** | |
| --- | --- | --- | --- | --- | --- | --- | --- |
|  | **Alive Patients** | **Deceased Patients** | **p-value** | **OR (95%CI)** | **p-value** | **HR (95%CI)** | **p-value** |
| **Imaging sub-cohort (n=231)** | | | | | | | |
| **Age** (years) | 28.82 | 27.93 | 0.5424 | 1.13 (1.03 - 1.27) | 0.022 | 1.02 (0.99 - 1.05) | 0.306 |
| **BMI** (kg/m^2^) | 25.40 | 23.46 | <0.001 | 1.05 (0.82 - 1.33) | 0.695 | 0.99 (0.90 - 1.09) | 0.827 |
| **LMI** (kg/height²) | 28.06 | 23.12 | <0.001 | 0.92 (0.81 - 1.03) | 0.157 | 0.99 (0.94 - 1.04) | 0.607 |
| **Risk Group** |  |  |  |  |  |  |  |
| Good (Ref) | 120 (77.9%) | 9 (13.0%) |  | 1.00 |  | 1.00 |  |
| Intermediate | 16 (10.4%) | 19 (27.5%) |  | 34.58 (5.37 - 333) | <0.001 | 5.45 (2.33 - 12.75) | <0.001 |
| Poor | 18 (11.7%) | 41 (59.4%) |  | 37.88 (5.04 - 454) | 0.001 | 6.61 (2.93 - 14.92) | <0.001 |
| **Histology** |  |  | <0.001* |  |  |  |  |
| Seminoma (Ref) | 61 (39.6%) | 10 (14.5%) |  | 1.00 |  | 1.00 |  |
| Non-Seminoma | 93 (60.4%) | 59 (85.5%) |  | 1.47 (0.19 -12.21) | 0.713 | 0.65 (0.28 - 1.49) | 0.309 |
| **Albumin** (g/dL) | 4.30 | 3.36 | <0.001 | 0.02 (0.00 - 0.09) | <0.001 | 0.21 (0.12 - 0.35) | <0.001 |
| **Glucose** (mg/dL) | 91.27 | 92.65 | 0.5262 | 0.99 (0.93 - 1.05) | 0.664 | 1.01 (0.99 - 1.02) | 0.447 |
| **Triglycerides** (mg/dL) | 212.02 | 123.38 | <0.001 | 0.99 (0.97 - 1.00) | 0.050 | 0.99(0.989 – 0.999) | 0.016 |
| **Total Cholesterol** (mg/dL) | 183.42 | 147.92 | <0.001 | 0.96 (0.93 - 0.99) | 0.019 | 0.99(0.982 - 0.998) | 0.010 |
| **HDL** (mg/dL) | 40.56 | 33.37 | <0.001 | 0.88 (0.80 - 0.95) | 0.005 | 0.96 (0.93 - 0.99) | 0.005 |
| **LDL** (mg/dL) | 112.85 | 101.81 | 0.004 | 1.02 (0.98 - 1.07) | 0.265 | 1.00 (0.99 - 1.02) | 0.668 |

**Abbreviations: OR**, Odds Ratio; **HR**, Hazard Ratio; **CI**, Confidence Interval; **BMI**, Body Mass Index; **LMI**, Lean Mass Index; **IGCCCG**, International Germ Cancer Collaborative Group; **HDL**, High-Density Lipoprotein; **LDL**, Low-Density Lipoprotein.

**Note:** This table presents the **initial exploratory analysis** using a **saturated multivariate model** that incorporates all available clinical, body composition, and metabolic variables simultaneously.

**Methodological Note:** In this high-dimensional model, the concurrent inclusion of biologically related variables (e.g., BMI and LMI; Histology and IGCCCG Risk Group; Total Cholesterol and LDL) results in **multicollinearity**, which attenuates the statistical significance of individual body composition markers. Consequently, optimized parsimonious models (separating BMI and LMI and prioritizing HDL) were constructed for the main analysis (Table 2) to accurately determine independent prognostic value.
