# Supplementary material for: Metabolic profile and skeletal muscle as predictors of survival in testicular germ cell tumors
Source: Oncologist. 2026 Apr 16;31(5):oyag072. doi: 10.1093/oncolo/oyag072 (PMC13092131; doi:10.1093/oncolo/oyag072)
Supplement: oyag072_Supplementary_Data [file oyag072_supplementary_data.zip › renamed_b0429.docx]

**Table 2.** Univariate and multivariate analysis of clinical, nutritional, and metabolic factors associated with mortality in patients with TGCT.

| **Variables** | **Logistic Regression** | | **Cox Regression** | | **Univariate Analysis** | | |
| --- | --- | --- | --- | --- | --- | --- | --- |
|  | **OR (95%CI)** | **p-value** | **HR (95%CI)** | **p-value** | **Alive Patients** | **Deceased Patients** | **p-value** |
| **Metabolic sub-cohort (n=586)** | | | | |  |  |  |
| **Age** (years) | 1.06 (1.02, 1.10) | 0.007 | 1.05 (1.03, 1.07) | <0.001 | 28.4 | 31.6 | 0.007 |
| **BMI** (kg/m^2^) | 0.95 (0.84, 0.97) | 0.05 | 0.91 (0.86, 0.97) | 0.006 | 25.11 | 23.73 | <0.001 |
| **Stage** | 2.04 (1.34, 3.19) | <0.001 | 1.40 (1.11, 1.76) | <0.001 | 1 | 2-3 | <0.001 |
| **Albumin** (g/dL) | 0.08 (0.03, 0.17) | <0.001 | 0.29 (0.20, 0.41) | <0.001 | 4.11 | 3.17 | <0.001 |
| **Glucose** (mg/dL) | 1.00 (0.98, 1.03) | 0.80 | 1.00 (0.99, 1.02) | 0.52 | 94.16 | 95.93 | 0.28 |
| **Triglycerides** (mg/dL) | 0.98 (0.97, 0.99) | <0.001 | 0.99 (0.99, 0.99) | 0.005 | 205.77 | 111.73 | <0.001 |
| **Total Cholesterol** (mg/dL) | 0.96 (0.94, 0.98) | <0.001 | 0.99 (0.98, 1.00) | 0.023 | 182.42 | 134.37 | <0.001 |
| **HDL** (mg/dL) | 0.85 (0.79, 0.89) | <0.001 | 0.95 (0.93, 0.97) | <0.001 | 40.42 | 31.62 | <0.001 |
| **LDL** (mg/dL) | 0.97 (0.97, 0.98) | 0.05 | 1.00 (0.99, 1.01) | 0.57 | 115.02 | 92.16 | <0.001 |
| **Imaging sub-cohort (n=231) BMI (kg/m^2^) analysis** | | | | |  |  |  |
| **Age** (years) | 1.05 (0.99–1.11) | 0.08 | 1.03 (1.00–1.05) | 0.027 |  |  |  |
| **BMI** / **LMI** | 0.81 (0.67–0.97) | 0.034 | 0.92 (0.84–0.99) | 0.047 |  |  |  |
| **Risk Group**  (IGCCCG) | 5.24 (2.85–10.6) | <0.001 | 2.11 (1.50–2.95) | <0.001 |  |  |  |
| **Albumin** (g/dL) | 0.03 (0.01–0.09) | <0.001 | 0.23 (0.15–0.36) | <0.001 |  |  |  |
| **HDL** (mg/dL) | 0.84 (0.78–0.90) | <0.001 | 0.94 (0.91–0.96) | <0.001 |  |  |  |
| **Imaging sub-cohort (n=231) LMI (kg/m^2^) analysis** | | | | |  |  |  |
| **Age** (years) | 1.04 (0.98–1.10) | 0.152 | 1.02 (0.99–1.04) | 0.145 |  |  |  |
| **BMI** / **LMI** | 0.89 (0.80–0.98) | 0.026 | 0.95 (0.90–0.99) | 0.047 |  |  |  |
| **Risk Group**  (IGCCCG) | 4.86 (2.65–9.74) | <0.001 | 2.09 (1.48–2.94) | <0.001 |  |  |  |
| **Albumin** (g/dL) | 0.03 (0.01–0.10) | <0.001 | 0.27 (0.18–0.42) | <0.001 |  |  |  |
| **HDL** (mg/dL) | 0.84 (0.77–0.90) | <0.001 | 0.94 (0.91–0.96) | <0.001 |  |  |  |

**Abbreviations:** **OR** Odds Ratio; **HR**, Hazard Ratio; **CI**, Confidence Interval; **BMI**, Body Mass Index; **LMI**, Lean Mass Index; **IGCCCG**, International Germ Cell Cancer Collaborative Group; **HDL**, High-Density Lipoprotein.

**Note:** The **Metabolic Sub-cohort (n=586)** section displays baseline characteristics stratified by vital status and univariate regression analysis for the full cohort. The **Imaging Sub-cohort (n=231)** section presents multivariate Logistic and Cox regression analyses. Two separate models were constructed to evaluate body composition: one including **BMI** and one including **LMI**. Both multivariate models are adjusted for Age, HDL, IGCCCG Risk Group, and Serum Albumin.
