# Supplementary material for: Metabolic profile and skeletal muscle as predictors of survival in testicular germ cell tumors
Source: Oncologist. 2026 Apr 16;31(5):oyag072. doi: 10.1093/oncolo/oyag072 (PMC13092131; doi:10.1093/oncolo/oyag072)
Supplement: oyag072_Supplementary_Data [file oyag072_supplementary_data.zip › renamed_bd68d.docx]

**Supplementary Table 4.** Baseline clinical and demographic characteristics of the full cohort, metabolic sub-cohort, and imaging sub-cohort in patients with TGCTs.

| Variable | Full  cohort (n=2755) | Metabolic  sub-cohort  (n=586) | Imaging  sub-cohort  (n=231) | p-value |
| --- | --- | --- | --- | --- |
| Age, years (median)  (Q1-Q3) | 27  (22-32) | 27  (22-34) | 26  (21-33.5) | 0.575 |
| BMI, kg/m^2^ (median)  (Q1-Q3) | 24.9  (22.6-27.7) | 24.0  (21.1-27) | 24.2  (23-27) | 0.201 |
| *Clinical Stage of Disease* | | | | |
| Stage III (distant metastasis) (n) | 543 | 183 | 98 | 0.086 |
| *Histological Classification* | | | | |
| Seminoma (n)  (%) | 994  (36.1%) | 194  (33.1%) | 79  (34.2%) | 0.360 |
| Non-seminoma (n)  (%) | 1761  (63.9%) | 392  (66.9%) | 152  (65.8%) | 0.360 |

Continuous variables are presented as medians with interquartile ranges defined as the 25th (Q1) to the 75th (Q3) percentiles (Q1-Q3).
