# Supplementary material for: Metabolic profile and skeletal muscle as predictors of survival in testicular germ cell tumors
Source: Oncologist. 2026 Apr 16;31(5):oyag072. doi: 10.1093/oncolo/oyag072 (PMC13092131; doi:10.1093/oncolo/oyag072)
Supplement: oyag072_Supplementary_Data [file oyag072_supplementary_data.zip › renamed_be1fa.docx]

**Supplementary Table 2. Bootstrap-Validated Performance Metrics of Multivariate Logistic Regression Models Predicting Mortality Risk in Patients with TGCT Stratified by Histology.**

| **Histological Subtype** | **Model Type** | **Predictors Included** | **AUC (95% CI)†** | **Sensitivity** | **Specificity** | **Accuracy** | **AIC** |
| --- | --- | --- | --- | --- | --- | --- | --- |
| **Patients with Non-Seminoma** | Complete | Age, BMI, LMI, Alb, Chol, HDL, LDL, Trig | 0.968 (0.934 – 0.992) | 0.925 | 0.9 | 0.914 | 58.33 |
|  | Reduced‡ | BMI, LMI, Albumin | 0.866 (0.781 – 0.938) | 0.887 | 0.8 | 0.849 | 87.48 |
| **Patients with Seminoma** | Complete | Age, BMI, LMI, Alb, Chol, HDL, LDL, Trig | 0.958 (0.859 – 1.00) | 0.875 | 1 | 0.969 | 31.41 |
|  | Reduced | BMI, LMI, Albumin | 0.922 (0.812 – 0.990) | 1 | 0.75 | 0.812 | 26.84 |

**Abbreviations:** AUC, Area Under the ROC Curve; CI, Confidence Interval; AIC, Akaike Information Criterion. **†** 95% Confidence Intervals were calculated using internal validation with bootstrapping (2,000 iterations). **‡** The NSGCT Reduced Model is selected as the primary prognostic model due to its balance of parsimony and robustness. **Note:** SGCT models show high performance but wide Confidence Intervals and unstable Odds Ratios due to the low number of mortality events in this subgroup
