# Supplementary material for: Metabolic profile and skeletal muscle as predictors of survival in testicular germ cell tumors
Source: Oncologist. 2026 Apr 16;31(5):oyag072. doi: 10.1093/oncolo/oyag072 (PMC13092131; doi:10.1093/oncolo/oyag072)
Supplement: oyag072_Supplementary_Data [file oyag072_supplementary_data.zip › renamed_d43f6.docx]

**Supplementary Table 8.** Metabolic-Nutritional Profiles of PCA-Based Clusters in Advanced Prognostic Groups: Intermediate/Poor-Risk Non-Seminoma and Intermediate-Risk Seminoma.

| **Markers/Parameters** | **Non-Seminoma^a^** | | **Seminoma^b*^** | | |  |
| --- | --- | --- | --- | --- | --- | --- |
|  | **High Metabolic-Nutritional Risk Profile (N=56) 5y OS= 14.9%** | **Low Metabolic-Nutritional Risk Profile (N=29) 5y OS= 68.1%** | | **High Metabolic-Nutritional Risk Profile (N=4) 5y OS= 50%** | **Low Metabolic-Nutritional Risk Profile (N=5) 5y OS= 30%** | |
| **BMI (kg/m²)** | 23.04 | 25.52 | | 21.26 | 25.3 | |
| **LMI** | 23.16 | 26.63 | | 21.23 | 27.87 | |
| **IGCCCG Risk Groups** | Poor | Poor | | Intermediate | Intermediate | |
| **Albumin (g/dL)** | 3.4 | 4.1 | | 3.62 | 3.7 | |
| **Glucose (mg/dL)** | 93.74 | 94.48 | | 83.75 | 84.2 | |
| **Total Cholesterol (mg/dL)** | 141.02 | 212.45 | | 170 | 190.2 | |
| **HDL (mg/dL)** | 32.45 | 42.48 | | 40.81 | 41 | |
| **LDL (mg/dL)** | 94.31 | 135.24 | | 100.42 | 111.98 | |
| **Triglycerides (mg/dL)** | 117.47 | 235.98 | | 82.8 | 201.12 | |
| **Age (years)** | 25.96 | 27.93 | | 27 | 37 | |

**^a^Patients with Non-Seminoma:** The High Metabolic-Nutritional Risk Profile (Cluster 1) exhibited a significantly depleted clinical status, characterized by lower albumin levels, hypolipidemia, and reduced muscle mass compared to the Low Metabolic-Nutritional Risk Profile (Cluster 2). Notably, these marked biological differences were observed despite both groups sharing identical IGCCCG prognostic scores ("Poor").

**^b^Patients with Seminoma:** The High Metabolic-Nutritional Risk Profile (Cluster 1) was distinguished by a compromised metabolic status with lower serum albumin. In contrast, the Low Metabolic-Nutritional Risk Profile (Cluster 2) maintained a more favorable metabolic and nutritional balance.

**Data Selection:** Analysis was restricted to IGCCCG Intermediate and Poor risk groups for Non-Seminoma, and the IGCCCG Intermediate risk group for Seminoma (the Poor risk category is not applicable for pure Seminoma).

*Subgroup analysis restricted to Intermediate-Risk Seminoma was performed but limited by the small sample size (N=9), preventing definitive clustering conclusions in this specific subset.
