# Supplementary material for: Metabolic profile and skeletal muscle as predictors of survival in testicular germ cell tumors
Source: Oncologist. 2026 Apr 16;31(5):oyag072. doi: 10.1093/oncolo/oyag072 (PMC13092131; doi:10.1093/oncolo/oyag072)
Supplement: oyag072_Supplementary_Data [file oyag072_supplementary_data.zip › renamed_de106.docx]

**Supplementary Table 7.** LMI by age group and survival status in patients with TGCTs.

| **Age Group**  **(years)** | **Alive**  **(n)** | **Deceased**  **(n)** | **Lean Mass Index (LMI)** | | **p-value** |
| --- | --- | --- | --- | --- | --- |
|  |  |  | **LMI (Alive)** | **LMI (Deceased)** |  |
| 15-19 | 23 | 18 | 26.05 | 23.12 | p = 0.038 |
| 20-29 | 72 | 31 | 28.76 | 23.25 | p < 0.0001 |
| 30-39 | 38 | 12 | 28.66 | 22.78 | p = 0.002 |
| >40 | 26 | 11 | 26.74 | 22.73 | p = 0.026 |

Age strata present mean LMI values for patients with available baseline CT imaging. Age stratification was used to adjust for physiological changes in muscle reserves, which decline gradually after the third decade of life.
