# Supplementary material for: Metabolic profile and skeletal muscle as predictors of survival in testicular germ cell tumors
Source: Oncologist. 2026 Apr 16;31(5):oyag072. doi: 10.1093/oncolo/oyag072 (PMC13092131; doi:10.1093/oncolo/oyag072)
Supplement: oyag072_Supplementary_Data [file oyag072_supplementary_data.zip › renamed_e0137.docx]

**Table 1.** Baseline demographic and clinical characteristics of patients with testicular germ cell tumors (TGCT) stratified by vital status.

| Variable | Total  (n = 2755) | Alive  (n = 2343) | Deceased  (n = 412) | p-value |
| --- | --- | --- | --- | --- |
| Age, years (median)  (Q1-Q3) | 27  (22-32) | 27  (22-32) | 26  (21-31) | 0.0307 |
| Body weight, kg (median)  (Q1-Q3) | 72  (64-80.5) | 72  (64-81.5) | 70  (61-78) | <0.001 |
| Height, cm (median)  (Q1-Q3) | 1.7  (1.65-1.74) | 1.7  (1.65-1.74) | 1.7  (1.64-1.73) | <0.001 |
| Body Mass Index (BMI), kg/m^2^ (median)  (Q1-Q3) | 24.9  (22.6-27.7) | 25.1  (22.7-28) | 24.2  (22.075-26.8) | <0.001 |
| *Clinical Stage of Disease^a^* | | | | |
| Stage I (localized)  (%) | 658 | 645  (27.5%) | 13  (3.2%) |  |
| Stage II (regional lymph node involvement)  (%) | 351 | 320  (13.6%) | 31  (7.5%) |  |
| Stage III (distant metastasis)  (%) | 543 | 363  (15.5%) | 180  (43.7%) |  |
| *Histological Classification* | | | | |
| Seminoma (n)  (%) | 994  (36.1%) | 860  (36.7%) | 134  (32.5%) | <0.001 |
| Non-seminoma (n)  (%) | 1761  (63.9%) | 1483  (63.3%) | 278  (67.5%) | <0.001 |
| *Non-seminoma Subtypes* | | | | |
| Pure Teratoma (n)  (%) | 93  (5.3%) | 76  (5.1%) | 17  (6.1%) |  |
| Choriocarcinoma (n)  (%) | 18  (1.0%) | 9  (0.6%) | 9  (3.2%) |  |
| Embryonal Carcinoma (n)  (%) | 49  (2.8%) | 45  (3.0%) | 4  (1.4%) |  |
| Yolk Sac Tumor (n)  (%) | 35  (2.0%) | 29  (2.0%) | 6  (2.2%) |  |
| Mixed (n)  (%) | 1566  (88.9%) | 1321  (89.1%) | 245  (88.1%) |  |

Continuous variables are presented as medians with interquartile ranges defined as the 25th (Q1) to the 75th (Q3) percentiles (Q1-Q3), and categorical variables are presented as counts with percentages. ᵃPercentages for clinical stage are calculated based on the subset of patients with available clinical staging information, using the AJCC 8th edition classification.
