# Supplementary figures and images for: Metabolic profile and skeletal muscle as predictors of survival in testicular germ cell tumors
Source: Oncologist. 2026 Apr 16;31(5):oyag072. doi: 10.1093/oncolo/oyag072 (PMC13092131; doi:10.1093/oncolo/oyag072)

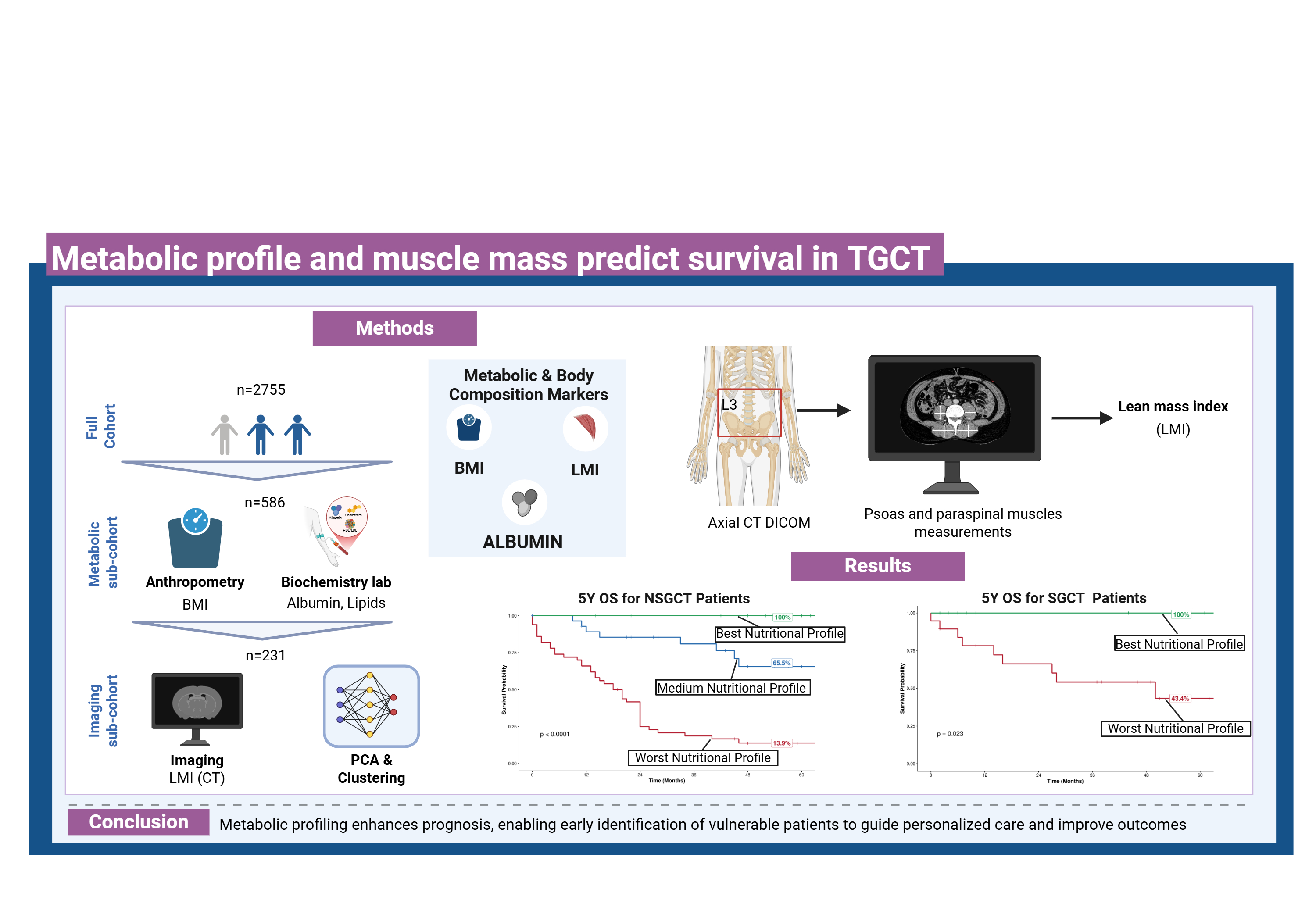

Supplement: oyag072_Supplementary_Data [file oyag072_supplementary_data.zip › renamed_64d9e.jpeg]

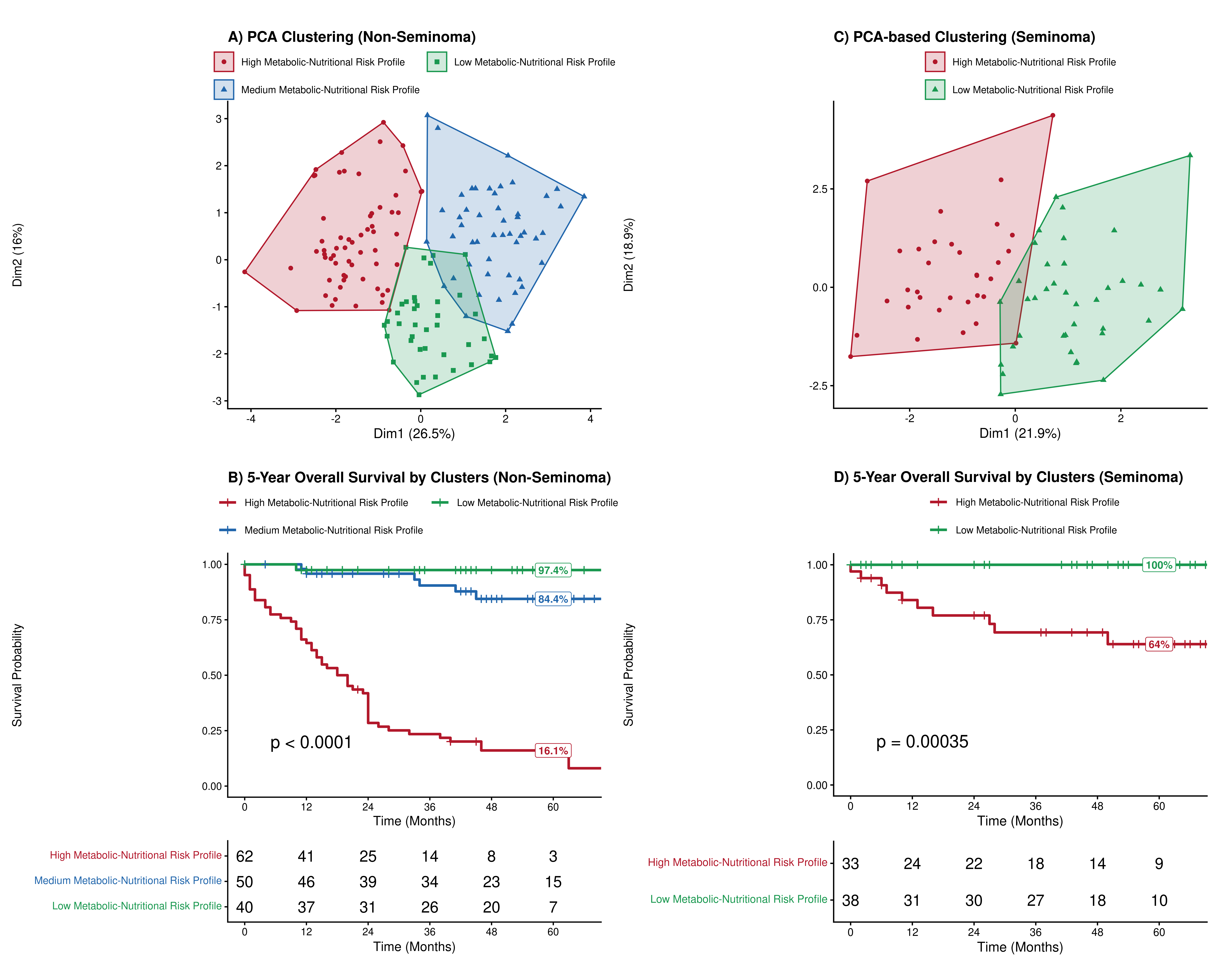

Supplement: oyag072_Supplementary_Data [file oyag072_supplementary_data.zip › renamed_7be29.tiff]

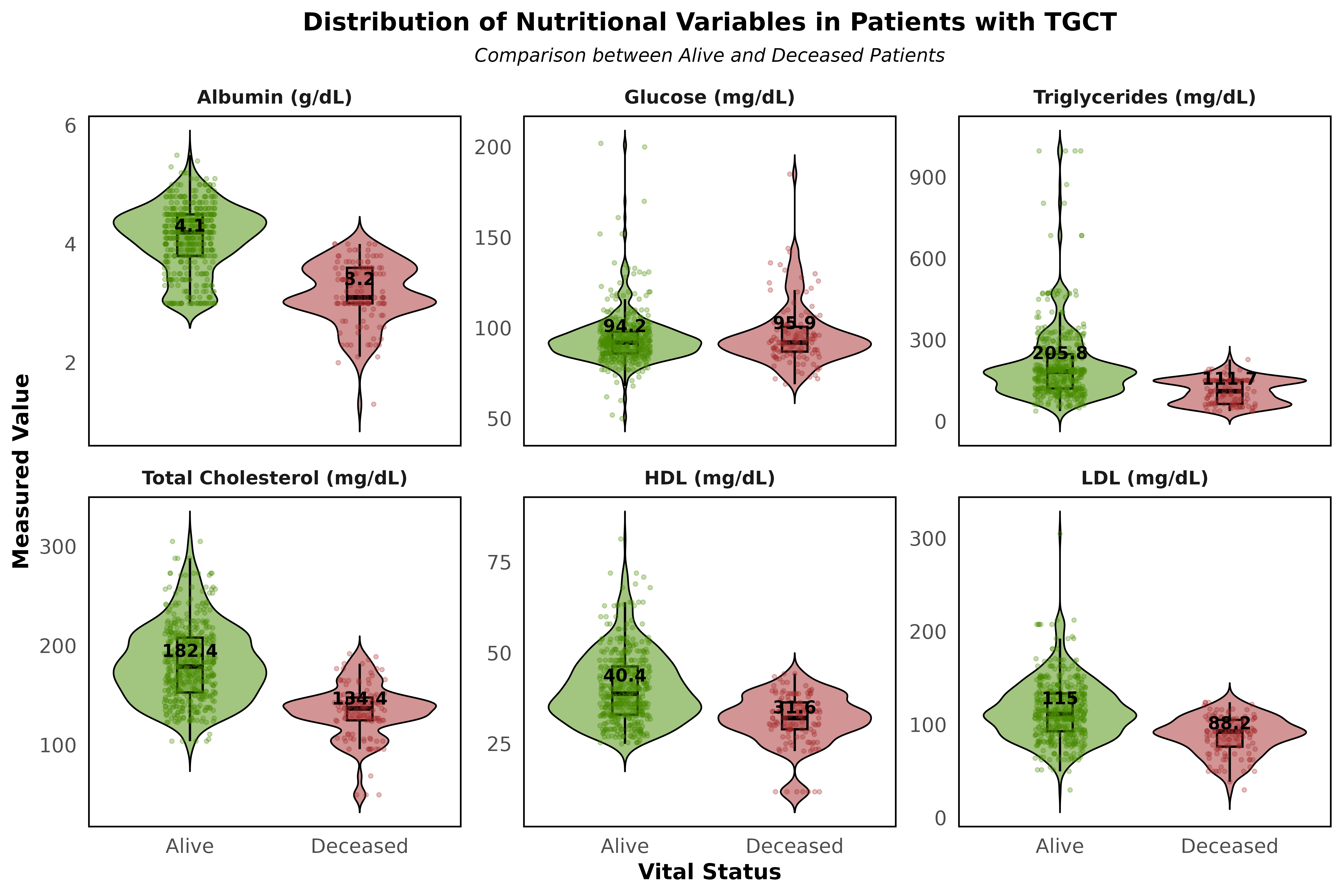

Supplement: oyag072_Supplementary_Data [file oyag072_supplementary_data.zip › renamed_160d6.tiff]

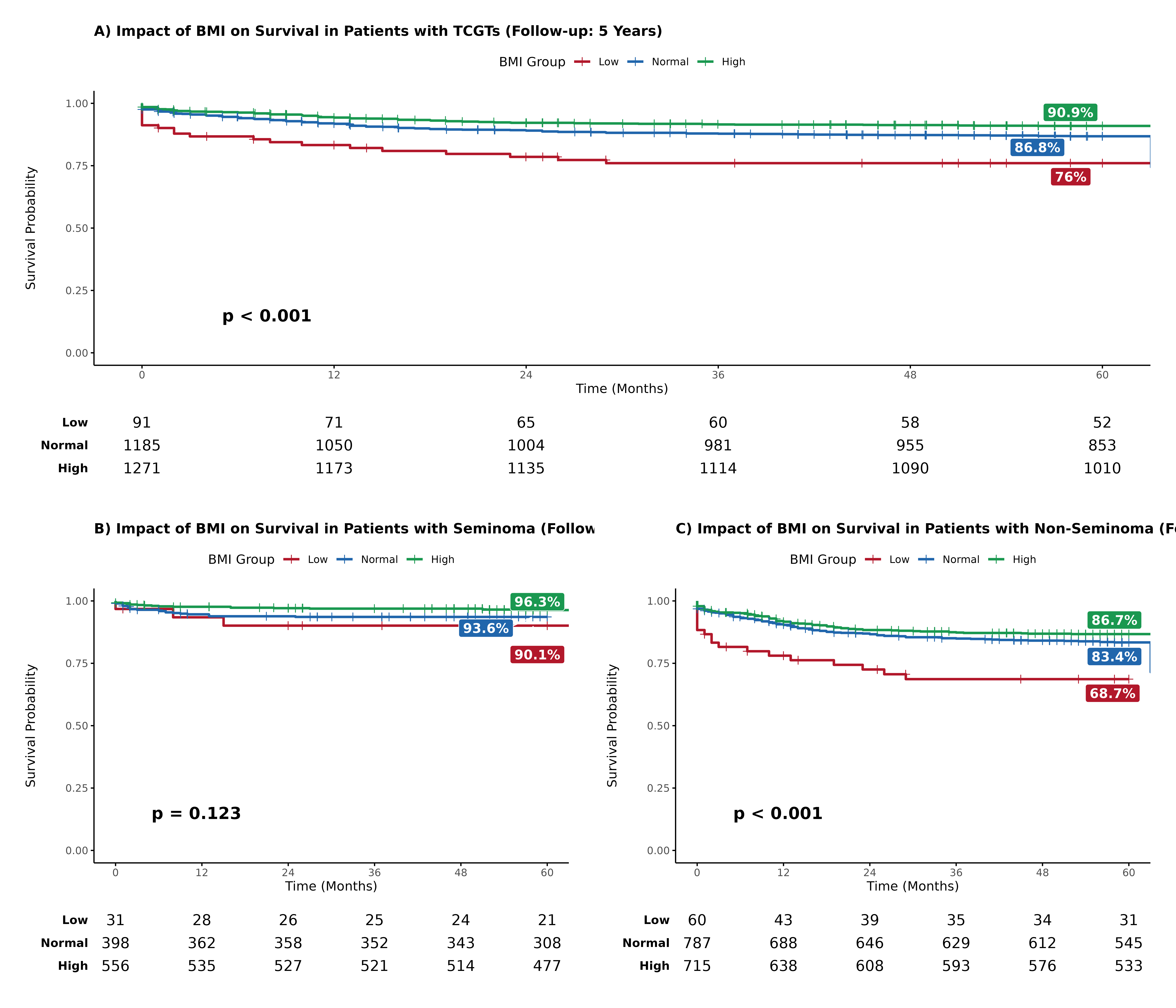

Supplement: oyag072_Supplementary_Data [file oyag072_supplementary_data.zip › renamed_161d9.tiff]
